# Comparative Analysis of Antioxidant Enzymes, Pigments, Phytochemicals, and Sensory Attributes in Different Phalsa (*Grewia asiatica* L.) Syrup Recipes

**DOI:** 10.1002/fsn3.71549

**Published:** 2026-02-22

**Authors:** Maida Arshad, Maryam Shabbir, Muhammad Amin, Sazada Siddiqui, Humaira Perveen, Muhammad Nafees, Hamza Niaz, Saqer S. Alotaibi, Muhammad Nasir Khan, Fahad Al‐Asmari, Tabarak Malik, Faisal Zulfiqar

**Affiliations:** ^1^ Department of Horticultural Sciences, Faculty of Agriculture and Environment The Islamia University of Bahawalpur Bahawalpur Pakistan; ^2^ Department of Biology, College of Science King Khalid University Abha Saudi Arabia; ^3^ Department of Food Science and Technology, Faculty of Agriculture and Environment The Islamia University of Bahawalpur Bahawalpur Pakistan; ^4^ Department of Biotechnology College of Science, Taif University Taif Saudi Arabia; ^5^ Renewable Energy and Environmental Technology Center University of Tabuk Tabuk Saudi Arabia; ^6^ Department of Science and Basic Studies, Applied College University of Tabuk Tabuk Saudi Arabia; ^7^ Department of Food and Nutrition Sciences, College of Agricultural and Food Sciences King Faisal University Al‐Ahsa Saudi Arabia; ^8^ Department of Biomedical Sciences, Institute of Health Jimma University Jimma Oromiya Ethiopia; ^9^ Division of Research and Development Lovely Professional University Phagwara Punjab India; ^10^ Korea University Seoul Republic of Korea

**Keywords:** *Grewia*, minor fruits, nutrition, quality, underutilized crops, value addition

## Abstract

The limited shelf life and short harvest season of phalsa ( *Grewia asiatica* L.) present challenges in the marketing of this valuable fruit, and this has prompted the focus on processing, value addition, and the exploration of alternative methods of consumption. The objective of this study was to develop two distinct recipes of phalsa syrup and compare them in terms of nutritional profile and sensory quality. The recipe 1 (500 g of fresh phalsa fruit boiled in 2 L water) and recipe 2 (750 g phalsa fruit boiled in 1 L water) were cooled, blended, strained and re‐boiled for 1 h. The ingredients of recipe 1 included 400 g sugar, 0.4 g sodium bicarbonate, and 30 mL of synthetic white vinegar while recipe 2 had 300 g sugar, 2.84 g black salt and 2 drops of synthetic red food color. The prepared syrups were cooled to room temperature, preserved in airtight bottles and stored at 5°C with 80%–85% RH for quality assessments. Both syrup types had very significant nutritional value and antioxidative properties. The activities of catalase (CAT) and superoxide dismutase (SOD) enzymes were greater in recipe 1 (17.23 and 13.77 U mg^−1^ respectively). The higher levels of pigments (0.57 mg 100 g^−1^ anthocyanins and 2.63 μg g^−1^ carotenoids), were detected in recipe 2. Regarding the phytochemical content, total soluble solids (TSS) were higher in recipe 2 (60.6 °Brix), while the recipe 1 had higher (191.7 mg L^−1^) total dissolved solids (TDS). The sensory evaluation indicated better color rating (7.9) for the recipe 2 with similar aroma, flavor and overall acceptability for both recipes. The color assessment indicated similar luminosity (L*) values (31.20 for recipe 1 and 32.38 for recipe 2), and positive a* and b* in both recipes with higher a* (redness) in recipe 2 (3.44) and more b* yellowness in recipe 1 (2.59). Overall, it was found that the higher pulp content in the phalsa fruit syrup improves the peroxidase activity, soluble solids and natural pigments; and the formulation differences affects biochemical and sensory quality of phalsa syrup.

## Graphical abstract

The short shelf life and limited harvest season of phalsa ( *Grewia asiatica* L.) necessitate processing and value addition for improved utilization. Two phalsa syrup formulations differing in pulp concentration and ingredients were developed and evaluated for nutritional, biochemical, and sensory quality. Recipe 1 showed higher antioxidant enzyme activities, with catalase and superoxide dismutase values of 17.23 and 13.77 U mg^−1^, respectively. Recipe 2 exhibited higher pigment content, total soluble solids (60.6 °Brix), and superior color acceptability. Overall, increased pulp content and formulation variations significantly influenced the biochemical and sensory attributes of phalsa syrup.

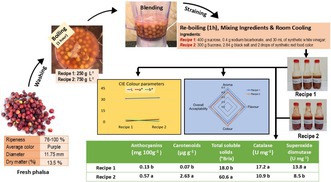

## 1 Introduction

*Grewia asiatica* , commonly known as phalsa, is a highly nutritive fruit. It is an excellent source of vitamin A and vitamin C and is rich in carbohydrates (6.8%–25.8%) and total sugars (5.73%–9.75%), with a protein composition of 1.5%, 0.9% fat, and 0.42%–2.5% acid (Sharma et al. 2024), mainly citric and malic acids, along with key minerals (iron, calcium, phosphorus, magnesium), bioactive compounds, and metabolites such as anthocyanins, flavonoids, tannins, and polyphenols.

The phalsa fruit contains significant medicinal properties (Akram et al. 2019). Its consumption provides a substantial amount of dietary fiber (Gochar et al. 2019), which aids in digestion, diarrhea, intestinal infection, cough, and jaundice treatment (Kaur et al. 2024) and helps maintain healthy cholesterol levels. Additionally, its high‐water content makes it a hydrating fruit, while its natural sugars offer a quick source of energy. The presence of antioxidants such as polyphenols and flavonoids contributes to the anti‐inflammatory and antioxidant properties. It also contains antihyperglycemic, radioprotective, hepatoprotective, antifungal, and antiviral properties. It is also used to treat conditions such as loss of appetite, typhus, acidity, dizziness, diarrhea, hypertension, and anorexia (Ray and Bala 2019). The phalsa fruit consumption also supports bone health, energy, and metabolism. It is also effective for improving skin health (Baraily 2021).

The phalsa fruit is highly perishable in nature, with a very limited shelf life of about 2 days only (Khan et al. 2019; Chinnaswamy et al. 2023) and is usually subjected to local marketing. Therefore, the processing of phalsa fruit is indispensable (Mehmood et al. 2020). Different thermal and nonthermal processing methods are used to preserve the shelf‐life of juice products (Nadeem et al. 2021). It is processed into jams, pies, chutneys (Zia‐Ul‐Haq et al. 2013) and ready‐to‐serve beverages such as juice (Chaturvedi et al. 2014) and squash (Rashid et al. 2021), contributing to its commercial appeal. The phalsa drinks with relatively high nutritional value can be prepared with minimal processing (Sinha et al. 2015). However, such beverages ferment quickly; therefore, preservatives are necessary to extend its shelf‐life (Tripathi 2009).

The fruit pulp‐based syrup development is an important option for value addition, long‐term preservation, postharvest loss reduction and shelf‐life extension in perishable fruits (Pinto et al. 2022). The phalsa fruit syrup may be a natural source of essential nutrients and bioactive compounds (Kaur et al. 2024). This study aimed to investigate the nutritional attributes of two different phalsa syrup recipes, including antioxidative enzymes, pigments, phytochemicals, and sensory properties.

## 2 Materials and Methods

### 2.1 Experimental Layout

**FIGURE 1:**
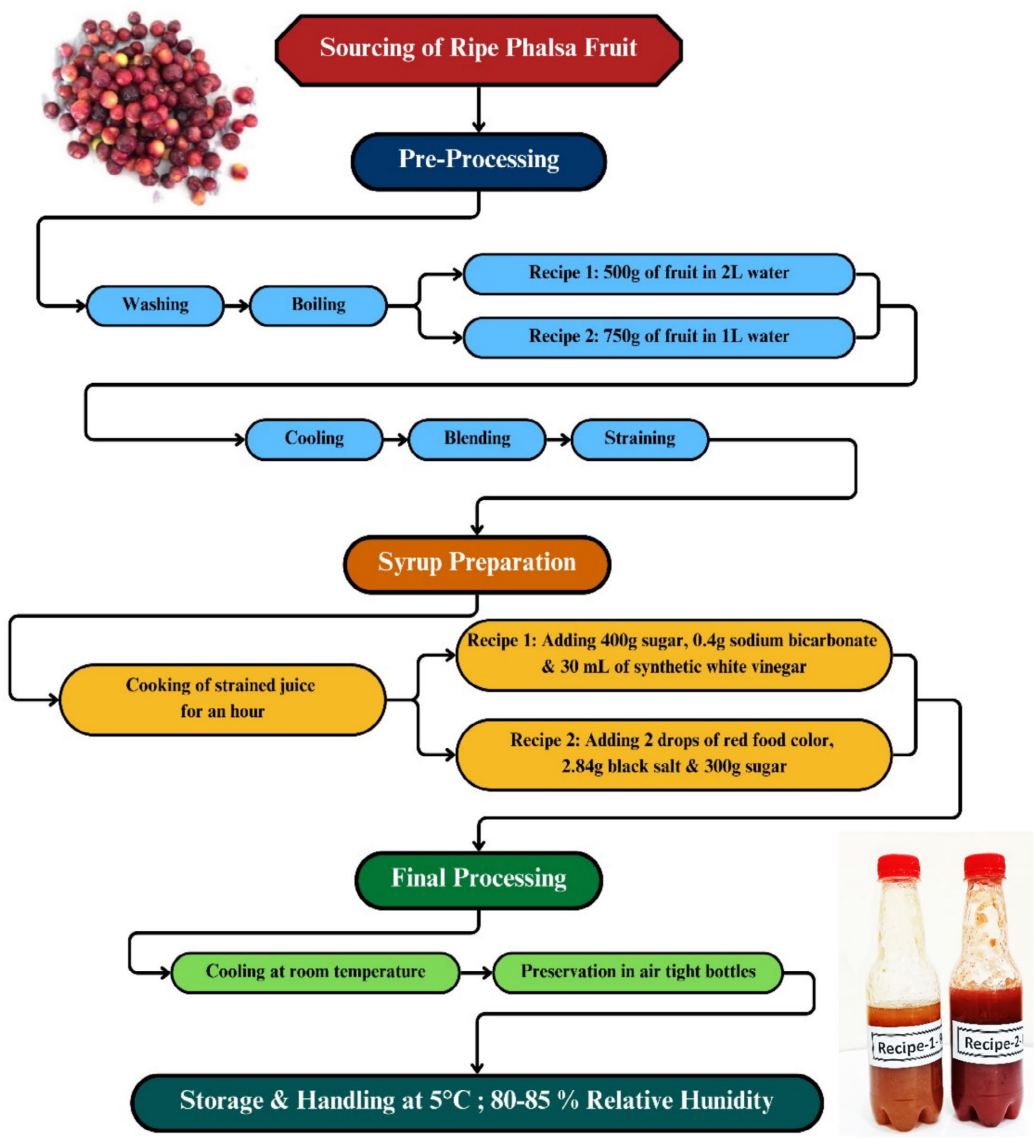
Processing procedures for phalsa syrup recipes.

The study was conducted under a completely randomized design (CRD) which included two recipes and three replications for the phalsa syrup preparations, with six number of experimental units (*n* = 6). Figure 1 represents the procedure of the experiment.

### 2.2 Fruit Sourcing and Syrup Preparation

Ripe fresh phalsa fruit (purple color, average diameter 11.75 mm) was purchased from the local retail market and subjected to the following procedures at the Postgraduate Research Laboratory, Department of Horticultural Sciences, The Islamia University of Bahawalpur, Pakistan, after careful washing.

#### 2.2.1 Recipe 1

500 g of phalsa fruit was dipped in 2 L of drinking water (Nestlé Pure Life), followed by boiling until the pulp turned whitish in color, indicating the breakdown of fruit components. The mixture was then cooled, blended, and strained to separate the juice. The strained juice was boiled for 1 h to make a concentrate with 400 g of sugar, 0.4 g of sodium bicarbonate, and 30 mL of synthetic white vinegar and allowed to cool to room temperature. After cooling, the syrup was preserved in an airtight container and kept in a refrigerator (5°C; 80%–85% RH).

#### 2.2.2 Recipe 2

750 g of fresh phalsa fruit was boiled in 1 L of water, cooled to room temperature, blended, and then strained. The strained juice was boiled over a high flame to concentrate it with the addition of 2 drops of synthetic red food color (Greens), 2.84 g of black salt, and 300 g of sugar. After cooling, the juice was stored in a refrigerator.

### 2.3 Data Collection

#### 2.3.1 Color Measurement (*L*, a*, b*)

The color was recorded via a portable color meter (10QC240641; FRU China) as per CIE *L*, a*, b* color system, where L* indicates the luminosity (0 = black, 100 = white), a* represents the redness (+) and greenness (−), and b* shows the yellowness (+) and blueness (−) (Bala and Barmanray 2019).

#### 2.3.2 Enzyme Assays

Different antioxidative enzymes, CAT, POX, and SOD, were assessed via the methods followed by Asghar et al. (2024). Two milliliters of phosphate buffer solution with a pH of 7.8 was used to homogenize the phalsa syrup with a mortar and pestle. After that, the mixture was centrifuged for 3 min at 4°C and 9000 rpm. The enzymatic activities were measured in the supernatant (enzyme extract). The falcon tube containing 100 μL of enzyme extract, 500 μL of phosphate buffer, 200 μL of methionine, 200 μL of Triton X, 100 μL of NBT, and 800 μL of distilled water was placed in a laminar flow hood for UV (ultraviolet) light exposure for 15 min to measure the amount of SOD present in each sample. Following UV removal, 100 μL of riboflavin was added, and a spectrophotometer was used to detect the absorbance at 560 nm. The CAT activity was estimated by adding 100 μL of enzyme extract to 100 μL of H_2_SO_4_ (5.9 mM) and measuring the absorbance at 240 nm. The POX activity was determined by adding 800 μL of phosphate buffer (pH 5) to 100 μL of H_2_O_2_ (40 mM) and 100 μL of guaiacol (20 mM) to the reaction mixture, followed by the addition of 100 μL of enzyme extract to 100 μL of the reaction mixture and measurement of the absorbance at 470 nm.

#### 2.3.3 Pigment Determination

Assessments were made for anthocyanins and carotenoids according to the methods described by Hassan et al. (2022). For the determination of anthocyanins, 1 g of phalsa syrup was placed in a Falcon tube along with 10 mL of the extraction mixture HCl + methanol (15:85) and incubated for 5 min at 25°C to measure the anthocyanin activity. After that, 200 μL of the supernatant was placed in a 96‐well plate, and the absorbances at 530, 620, and 650 nm were measured. The anthocyanin calculations were made as ΔA g^−1^ FW = (A530–A620)—0.1 (A650–A620), where A530, A620, and A650 are absorbance readings at specific wavelengths (nm). The assessment of total carotenoids was performed by placing 200 μL of the supernatant in a 96‐well plate, and the absorbances at 662, 645, and 470 nm were recorded. The carotenoid content was calculated as (1000A_470_‐1.90C_a_‐63.14C_b_)/214, where, A_470_ is the absorbance readings at specific wavelengths (nm), C_a_ stands for chlorophyll *a* and C_b_ for chlorophyll *b*.

#### 2.3.4 Phytochemical Analyses

The studied phytochemical activities included TSS, pH, TDS, and protein. The TSS (°Brix) was calculated via a digital refractive index (Model 01502B, ATC, China). A digital pH meter [pH‐009 (I)A] was used to measure the pH. A TDS meter (TDS/EC‐PRO, YIYEGO, China) was used to measure the TDS (mg/L). To estimate the protein content, 40 μL of enzyme extract was added to 16 μL of Bradford reagent, and the absorbance at 595 nm was determined (Hassan et al. 2022).

#### 2.3.5 Sensory Evaluation

Sensory evaluation comprised the judgment of various attributes (color, flavor, aroma, and overall acceptability) of phalsa syrup by a panel of 10 judges via a hedonic scale (Addo‐Preko et al. 2023).

### 2.4 Statistical Analysis

The data were subjected to statistical analysis (analysis of variance, least significant difference test) at the 5% level of significance (0.05 *p* value) via Statistix 8.1 software.

## 3 Results and Discussion

### 3.1 Color (L*, a*, b*)

**FIGURE 2:**
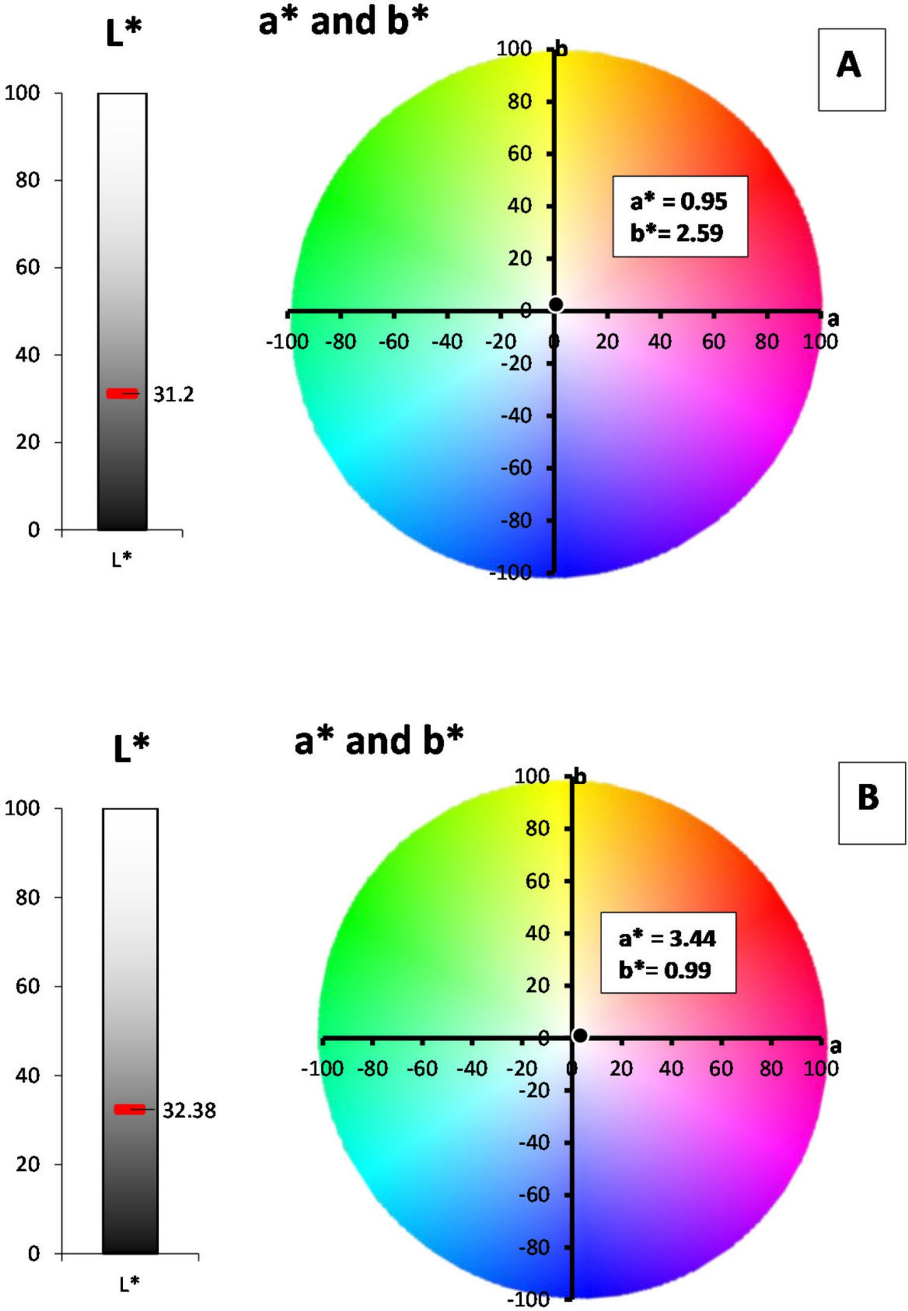
Color kinetics of Recipe 1 (A) and Recipe 2 (B).

Both recipes were dark in color due to their low luminosity (L*) values (31.20 for recipe 1 and 32.38 for recipe 2), which were statistically similar for both recipes (Figure 2). Moreover, in both recipes, a* and b* were positive (greater than 0), with red and yellow hues. The significantly higher a* value in recipe 2 (3.44) indicated more redness than in recipe 1 (0.95), whereas b* was significantly greater in recipe 1 (2.59), indicating more yellowness than in recipe 2 (0.99).

The lower luminosity values can be attributed to the release of water from the cellulose matrix due to thermal treatment, which also increased the viscosity of the syrup (Estrada‐Beltrán et al. 2024).

### 3.2 Antioxidative Enzymes

**FIGURE 3:**
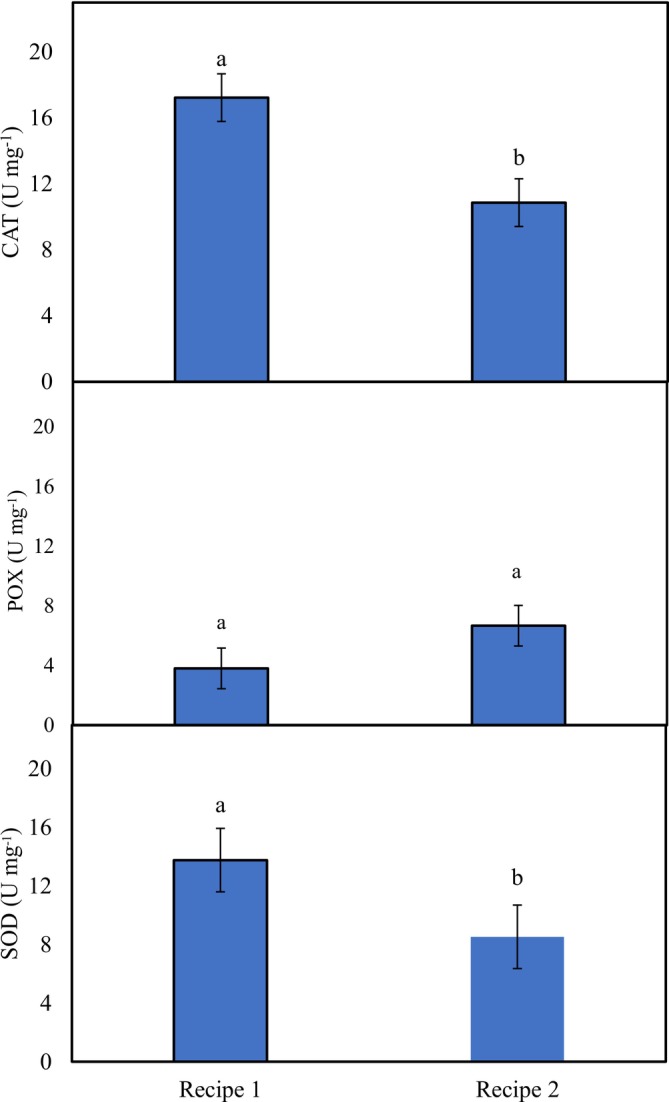
Activities of antioxidative enzymes in phalsa syrups (SE_(CAT)_ ± 1.44°C3, SE_(POX)_ ± 0.2404, SE_(SOD)_ ± 0.4989; *p* < 0.05). Different letters show significant difference among treatments.

Significant variation was found in the activities of CAT and SOD in the studied phalsa syrup recipes which were greater in recipe‐1 (17.23 and 13.77 U mg^−1^ respectively), whereas POX activity was similar among both recipes (Figure 3).

The antioxidative enzymes play a very important role in the human body by fighting reactive oxygen species and minimizing oxidative stress (Krishnamurthy and Wadhwani 2012; Adwas et al. 2019). The results of this study indicated that both phalsa syrups can be utilized for improving human health and reducing oxidative stress due to the presence of antioxidative enzymes. However, there is variation in the enzymatic attributes and antioxidative properties, which depend upon the processing method and organic acids (Abbès et al. 2013), type and characteristics of the raw materials (Yilmaz et al. 2020), maturity stage at harvest (Cao et al. 2019), and harvest period (Kunitake et al. 2014).

### 3.3 Pigments

**FIGURE 4:**
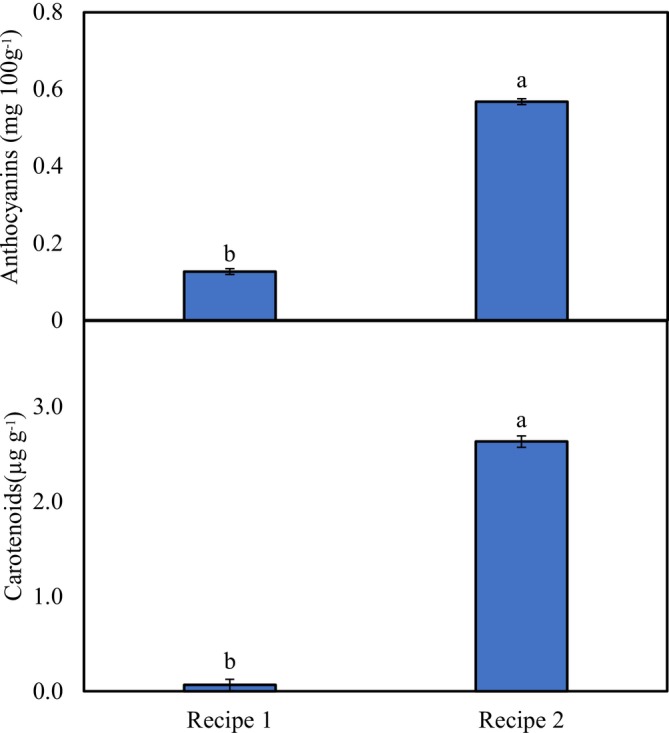
Activities of pigments in phalsa syrups (SE_(anthocyanin)_ ± 7.557, SE_(carotenoids)_ ± 0.0603; *p* < 0.05). Different letters show significant difference among treatments.

With respect to the activities of anthocyanin and carotenoids in the examined phalsa syrup recipes, significant variation was observed in both recipes, with higher values in recipe 2 (0.57 mg 100 g^−1^ anthocyanins and 2.63 μg g^−1^ carotenoids) (Figure 4).

Anthocyanins are pigments that exist naturally in various fruits, vegetables and flowers and have several positive effects on human health (Mattioli et al. 2020). Many factors, including temperature and light (Talpur et al. 2017), influence stability and may cause deterioration. In this study, the relatively high anthocyanin content in recipe‐2 seems to be the result of additive color.

Carotenoids are particularly prone to oxidation which can result in color changes and influence sensory properties (Tomlins et al. 2012). The stability of carotenoids in food products is affected by several factors. The significant difference in carotenoid content between the two syrup recipes indicates the variation in the stability and chemical properties of the two recipes (Yahia et al. 2017) during postharvest handling (Dias et al. 2014). Moreover as observed by Rodríguez‐Roque et al. (2016) the variation in the carotenoid content of fruit juice‐based beverages is associated with processing type.

### 3.4 Phytochemicals

**FIGURE 5:**
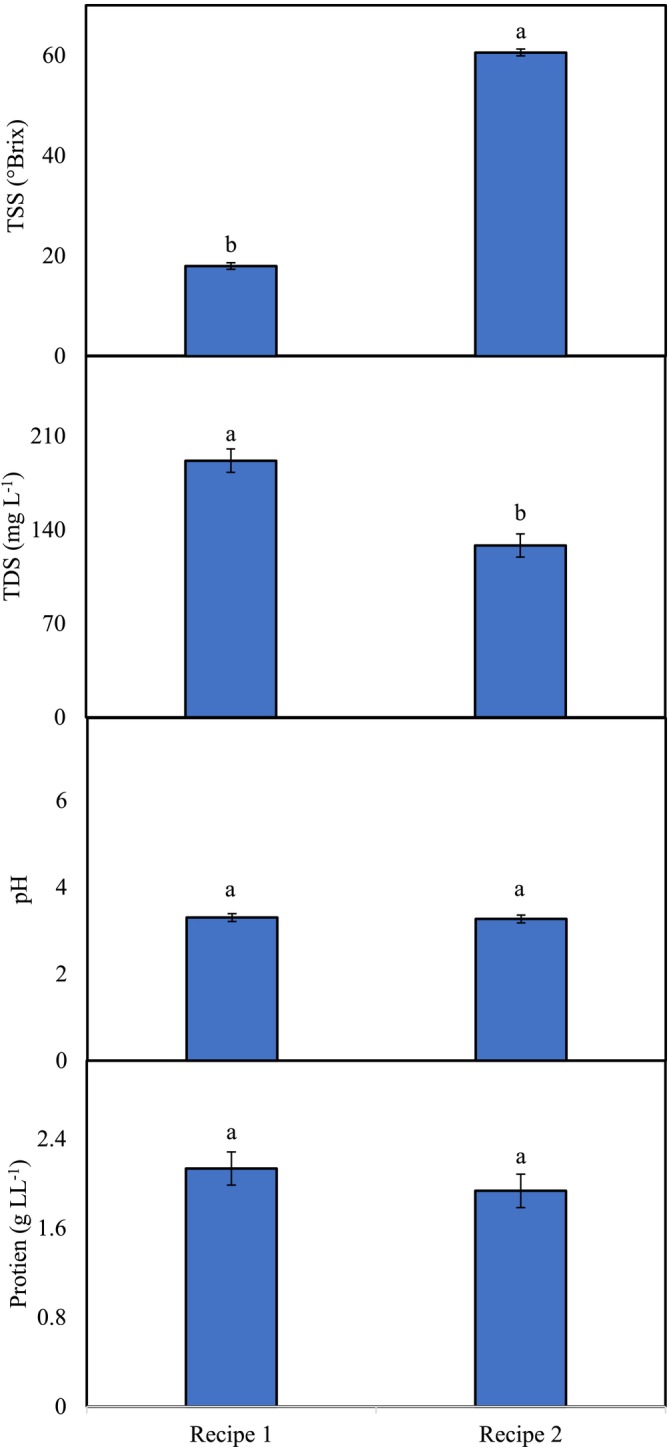
Activities of phytochemicals in phalsa syrups (SE_(protein)_ ± 0.1491, SE_(pH)_ ± 0.0882; *p* < 0.05; SE_(TSS)_ ± 0.6658, SE_(TDS)_ ± 8.6875; *p* > 0.05). Different letters show significant difference among treatments.

Significant variations in the activities of TSS and TDS were noted among the phalsa syrup recipes (Figure 5). The TSS content was greater in recipe‐2 (60.6 °Brix) than in recipe‐1 (18.0 °Brix), indicating a greater concentration of sugar and other solids and greater water evaporation during syrup heating. TDS values, while related to TSS values, include not only sugars but also organic acids, minerals, and other soluble components (Gbarakoro et al. 2020). The differences observed among the studied syrups reflect the effects of ingredient addition and variations in water content. The pH and protein content remained similar in both recipes (Figure 5). The low pH values in both recipes indicate microbial safety and shelf stability (Tribst et al. 2009), with good quality due to protein retention.

### 3.5 Sensory Attributes in Different Phalsa Syrup Recipes

**FIGURE 6:**
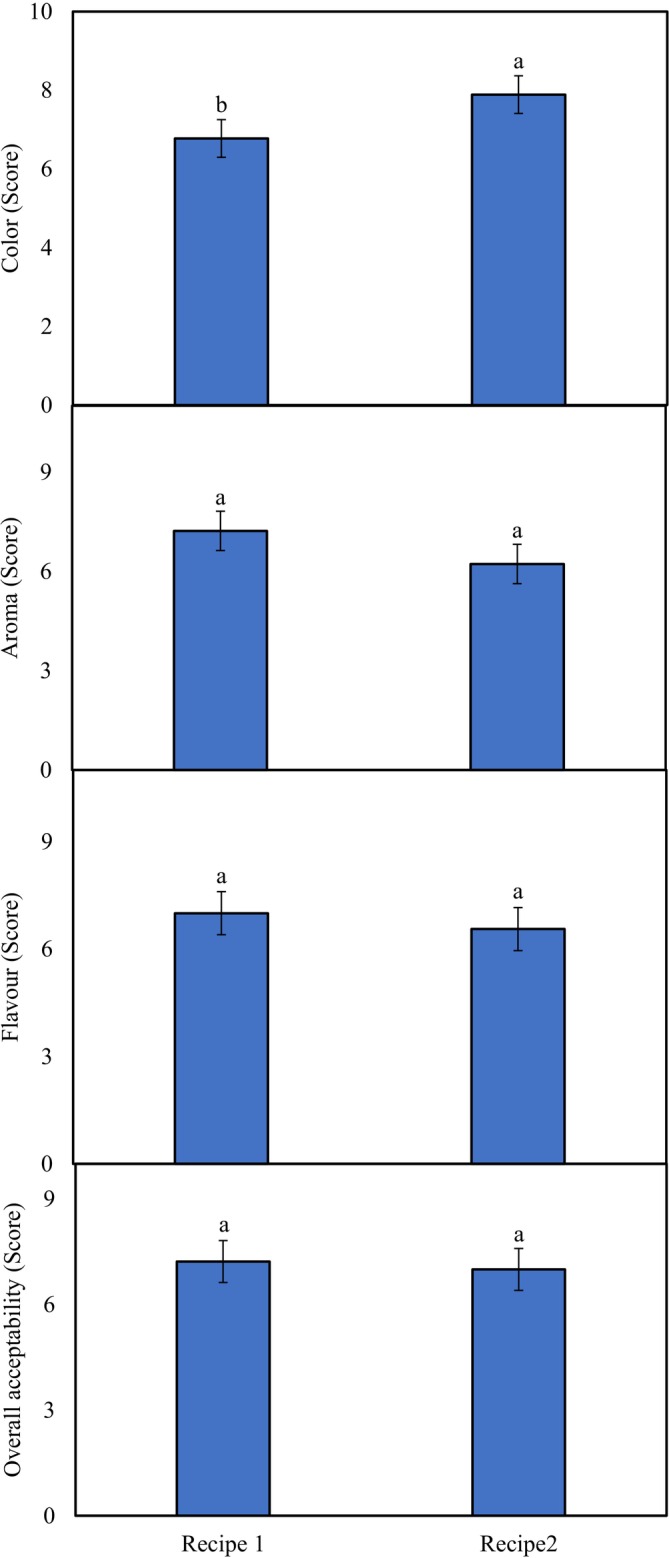
Activities of sensory attributes in phalsa syrups [SE_(color)_ ± 0.4779; *p* > 0.05; SE_(aroma)_ ± 0.5906, SE_(flavor)_ ± 0.6035, SE_(overall)_ ± 0.5958; *p* < 0.05]. Different letters show significant difference among treatments.

A significant difference was noted in the color of the studied phalsa syrup recipes, with a higher rank for recipe 2 (7.9 score) than in recipe 1 (6.8 score). The vibrant colors found in fruits are primarily due to plant pigments, such as anthocyanins (Figure 6), which are present in fruits (Pangotra et al. 2018) and may be due to the higher soluble solid content and denser red pigments in the juice (Zahir et al. 2021). The greater color in recipe 2 is linked with higher fruit content, leading to more anthocyanins (the main color compounds of phalsa). Moreover, the addition of food‐grade red also improved the color of recipe 2, thereby improving consumer acceptance. The aroma, flavor, and overall acceptance of both recipes were similar (Figure 6), which indicates the similarity between the two recipes in terms of these organoleptic properties.

## 4 Conclusions

The results of this investigation revealed the presence of antioxidants (enzymes and pigments) and biochemicals in the phalsa syrup, with good organoleptic scores. Among the two studied phalsa syrup recipes, REC_2_ was better due to greater peroxidase activity, higher values of pigments (anthocyanins and carotenoids), and higher TSS and color values. Future investigations should focus on evaluating the pilot‐scale market response, consumer sensory acceptance, microbial stability, and shelf‐life optimization of phalsa syrup.

## Author Contributions

**Maida Arshad:** conceptualization, methodology, investigation, writing – original draft. **Fahad Al‐Asmari:** writing – review and editing. **Muhammad Nafees:** visualization, validation. **Hamza Niaz:** visualization, validation. **Humaira Perveen:** conceptualization, methodology, investigation, writing – original draft. **Saqer S. Alotaibi:** data curation, formal analysis, writing – original draft. **Faisal Zulfiqar:** writing – review and editing, project administration, resources. **Tabarak Malik:** writing – review and editing, supervision. **Muhammad Nasir Khan:** formal analysis, data curation, writing – original draft. **Maryam Shabbir:** conceptualization, methodology, investigation, writing – original draft. **Sazada Siddiqui:** writing – review and editing, data curation, visualization. **Muhammad Amin:** conceptualization, investigation, methodology, writing – original draft.

## Funding

The authors extend their appreciation to the Deanship of Research and Graduate Studies at King Khalid University for funding this work through Large Group Project under grant number RGP2/89/46.

## Conflicts of Interest

The authors declare no conflicts of interest.

## Data Availability

The data that support the findings of this study are available from the corresponding author upon reasonable request.
